# Hopes and fears regarding care robots: Content analysis of newspapers in East Asia and Western Europe, 2001–2020

**DOI:** 10.3389/fresc.2022.1019089

**Published:** 2022-12-08

**Authors:** N. Kodate, Y. Maeda, B. Hauray, M. Tsujimura, W. C. H. Chan, H. Mannan, W. Yu, S. Dalgalarrondo, M. C. Cheung, A. Yumoto, S. Suwa, S. Donnelly, N. Sakata, D. O’Shea, K. Obayashi, S. Masuyama

**Affiliations:** ^1^ School of Social Policy, Social Work and Social Justice, University College Dublin, Dublin, Ireland; ^2^ Public Policy Research Center, Hokkaido University, Sapporo, Japan; ^3^ La Fondation France-Japon, School for Advanced Studies in the Social Sciences (EHESS), Paris, France; ^4^ Institute for Future Initiatives, University of Tokyo, Tokyo, Japan; ^5^ Universal Accessibility and Ageing Research Centre, Nishitokyo, Japan; ^6^ Faculty of Business, Technological University Dublin Dublin, Ireland; ^7^ Institut de recherche interdisciplinaire sur les enjeux sociaux, School for Advanced Studies in the Social Sciences (EHESS), Paris, France; ^8^ French National Institute of Health and Medical Research (INSERM), Paris, France; ^9^ School of Nursing, Shiga University of Medical Science, Otsu, Japan; ^10^ Department of Social Work, The Chinese University of Hong Kong, Hong Kong, Hong Kong SAR, China; ^11^ School of Nursing, Midwifery and Health Systems, University College Dublin, Dublin, Ireland; ^12^ Flame University, Pune, India; ^13^ Center for Frontier Medical Engineering, Chiba University, Chiba, Japan; ^14^ French National Centre for Scientific Research (CNRS), Paris, France; ^15^ Graduate School of Nursing, Chiba University, Chiba, Japan; ^16^ Center for Information and Communication Technology, Dokkyo Medical University, Mibu, Japan; ^17^ St Vincent’s University Hospital, Dublin, Ireland; ^18^ Royal College of Physicians of Ireland, Dublin, Ireland; ^19^ Faculty of Healthcare Management, Nihon Fukushi University, Mihama, Japan; ^20^ Social Welfare Corporation Tokyo Seishin-kai, Nishitokyo, Japan; ^21^ Traveler’s Medical Center, Tokyo Medical University, Tokyo, Japan

**Keywords:** robot, welfare technology, Disability, social care, public perception, Social constructivism, Asia, Europe

## Abstract

**Background:**

As a type of welfare technology, care robotics is now widely seen as a potential aide to rehabilitation, increasing independence and enhancing the wellbeing of people with disabilities and older adults. Research into and development of care robots have both been vigorously promoted in North America, Europe and Asia, and the competition for technological advancement in robotics is becoming fierce. AI ethics and policy guidelines are being established. However, there are still differences in attitudes and perceptions, as well as national policies regarding this type of welfare technology. Moreover, despite the anticipated usefulness, it is believed that progress has been slow in the diffusion of care robots.

**Purpose:**

In order to explore how public discourses support technological innovation, such as care robots, while preparing society for potential risks and impact, we sought to ascertain whether public discourse on care robots varies from region to region. For example, what are the hopes and promises associated with care robots and what are the concerns?

**Methods:**

To address these questions, this article explored how care robots have been portrayed in five major broadsheet newspapers in five jurisdictions in Asia and Europe (France, Great Britain, Hong Kong SAR, Ireland and Japan). We obtained 545 articles for the period between January 2001 and September 2020, more than half of which originated in Japan. A thematic analysis was conducted of these articles written in four languages (Chinese, English, French and Japanese).

**Results:**

Positive and negative narratives were teased out, alongside other key prominent themes identified, such as Japan as the land of robots, the pandemic, and the impact of robots on the economy. As the number of robot-related articles grew from the year 2012 onwards, narratives became more nuanced in European newspapers, but not in Asian ones. Furthermore, recent articles began to address the social and relational impact of care robots, while providing concrete examples of improvements in the quality of life for users. Further careful examination will be necessary in the future in order to establish the impact of robotics use in rehabilitation for people with disabilities, older adults, their carers and society at large.

## 1. Introduction

**Figure 1:**
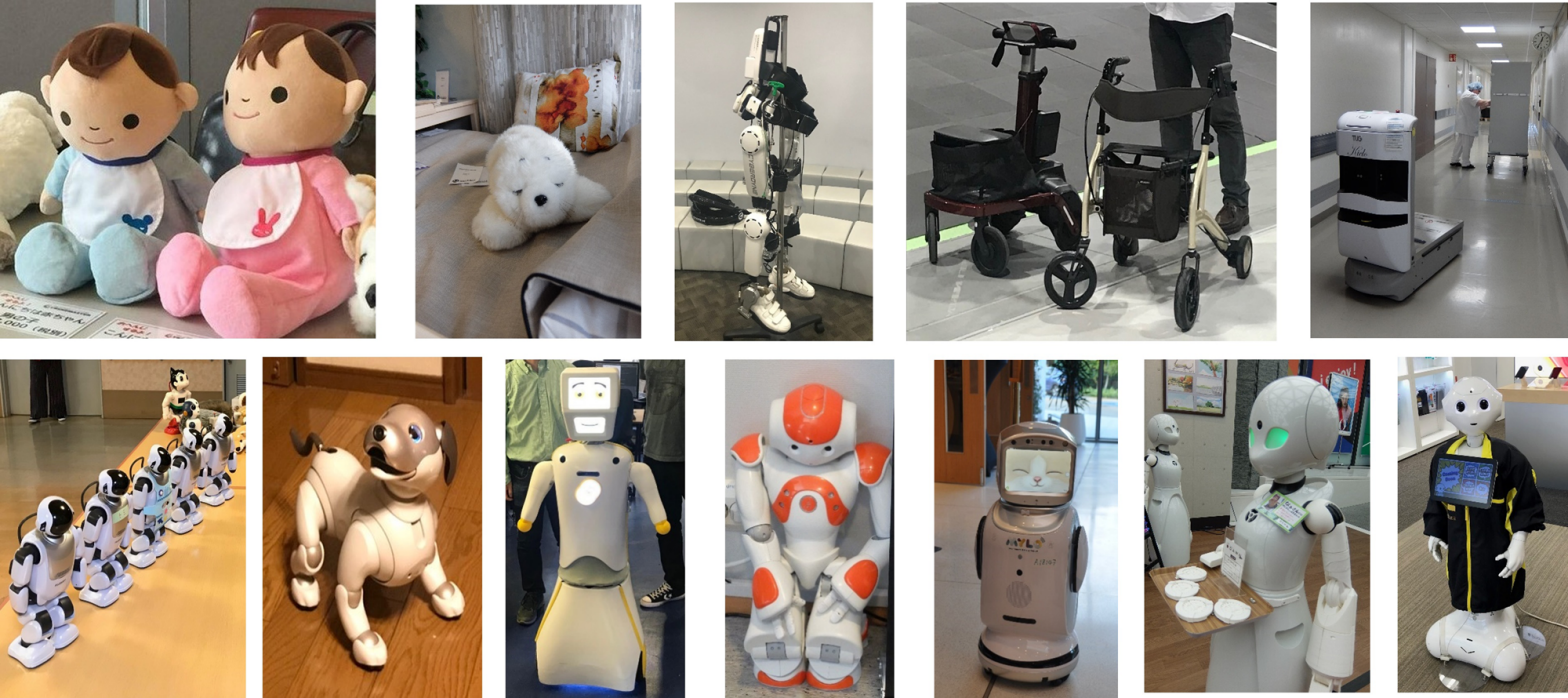
A variety of care robots that are in use in East Asia and Western Europe (photos by Kodate).

Assistive technologies (ATs), often dubbed as welfare technologies (WTs), are designed to promote and support the well-being of people in need, by enabling them to live a healthy, productive, and independent life (1). The research field of ATs has been evolving over a long period of time, with robots viewed as instruments, and device to “increase, maintain or improve functional capabilities of individuals with disabilities” (2). Article 26 of the United Nations Convention on the Rights of Persons with Disabilities (UNCRPD) maintains that “States Parties shall promote the availability, knowledge and use of assistive devices and technologies, designed for persons with disabilities, as they relate to habilitation and rehabilitation” (3). Care robots (see Figure 1 for examples) are now seen as a variant of ATs, and this area is growing rapidly (4). In May 2022, the World Health Organization (WHO) and the United Nations Children’s Fund (UNICEF) jointly published “The Global Report on Assistive Technology,” in which robotics is highlighted as “one of the most rapidly developing technologies” (5). A robot is an intelligent mechanical system with three main functions (detecting, assessing, and acting on the information), and has been introduced to rehabilitation (6) and telework for people with disabilities in order to enhance social inclusion (7). While research into and development of care robots have been vigorously promoted in North America, Europe and Asia, there are still differences in attitudes and perceptions, as well as national policies regarding care robots (8–10).

### 1.1. Technology in global society and care robots

As Max Weber noted, modernization and the development of capitalism in the West went hand in hand with technological advances (11). In social scientific inquiries, there are two opposing views about the role of technology in society. One view is that technology determines the development of social structure and cultural values (“technical determinism”), and the other view is that human actions including collective future visions and public discourse shape innovative technologies (“social construction of technology”), not the other way around (12,13).

Each epoch has its technological reference (14), and in our time, a social robot can provide a great example to test these two opposing perspectives. Currently, there is a strong trend in the discourse and policy communities, which supports the argument that robots will provide a solution to social and economic challenges associated with aging (15,16).

In domains such as manufacturing (e.g. Unimate), warfare (e.g. iRobot PackBot) and medicine (e.g. da Vinci surgical system, IBM’s Watson), the usefulness and merits of using robots have been emphasized and in part accepted (17,18). However, in public discourse around care settings, the dichotomy of “cold technologies vs. warm care” (19,20) has been a frequently-used narrative, with ethical concerns having been raised (4). This is supported by the deep-rooted binary view of “robots vs. humans” (21).

In popular culture, robots and cyborgs have been depicted as both heroes/ heroines and villains in cinema, anime, and PlayStation VR (17). In recent years, a series of theater plays featuring robots have been shown in Japan, Taiwan and elsewhere (22). These experiments certainly test our imaginations and force us to think about human-robot interactions. Fischer proposes an emerging research agenda (theorizing science, technology and society (STS) from Asia), stressing the potential importance of East and Southeast Asia as “strategic locales or sites of cultural critique and materials for new theory construction” in the STS domain (23). It has been reported that in Taiwan health professionals’ attitudes toward care robots have been positive (24). Japan in particular has a reputation for its “robotics culture” (25–29), and a survey conducted in 2015 in Japan indicated high levels of willingness among older respondents to incorporate a robot into their care (30).

On the other hand, two recent surveys carried out in Finland suggest that people have negative views towards the use of robots in older people’s care (8,31). Therefore, it can be assumed that public perceptions towards care robots may differ from culture to culture. Since the publication of the study by Nisbett et al. (32), the holistic-versus-analytic-cognition divide between East Asians and Westerners has been contested (33). A cross-regional comparison between Europe and Asia merits our investigation.

Furthermore, some previous studies (9,12) analyzed how care robots are perceived by the general public and by users, and how their perceptions, actual use and national policy can be interconnected or disconnected. The development and diffusion of innovative technology such as robots or automated vehicles are used to mobilize national resources and promote the economic growth of certain countries (e.g. techno-nationalism) (34,35). Techno-nationalism can be defined as a concept which “links technological innovation and capabilities directly to a nation’s national security, economic prosperity and social stability” (36).

From this standpoint, there is an interesting question as to the way in which public discourse in different jurisdictions is structured and how that can influence research and development of social robots and government policy concerning them. The seemingly universal idea of a “silver economy” in the era of a global aging population may exhibit some regional/national differences.

### 1.2. Research into and development of robots and government policies in Asia and Europe

Despite possible differences in attitudes and perceptions, research into and development of care robots have been promoted in both Europe and Asia. In the European Union, care robots have been developed as an assistive technology to tackle aging issues. Policy initiatives including Industry 4.0 (European Parliament, 2016) and SPARC (The Partnership for Robotics in Europe, 2016) supported the development of robots such as Robot-Era, Care-O-bot and Giraffe (37). In terms of robot density (the number of robots per 10,000 workers in the manufacturing industry), regionally, Asia and Australia come first with 134 units, followed by Europe (123 units) and the Americas (111) (38). Nationally, in Europe, Germany ranked 4th worldwide (371 units), followed by Sweden, Denmark and Italy. In Germany, the Federal Ministry of Education and Research (BMBF) has been funding research and development projects for robotic support of care under the banner of “Robotic Systems for Care” (Robotische Systeme für die Pflege) (BMBF n.d.). An Irish robot called Stevie made the cover of Time magazine. In Denmark, a feeding robot Bestic was implemented in care homes under the auspices of the government and local authorities (39). In advance of active policy implementation in Denmark, the Danish Council on Ethics published a report, “Social Robots: Opinion of the Council of Ethics” (40). In France, the robotics Research group (GdR Robotique/Groupement de Recherche Robotique) was created in CNRS (the National Centre for Scientific Research) in 2007, and there are a few leading firms such as Blue Frog Robotics. The above-mentioned robot density in France (194) is higher than the world average (126 robots per 10,000 workers), while the UK (101) and Ireland were below the average (38).

In Asian regions such as China, South Korea, Singapore, Hong Kong SAR and Taiwan, there has also been a strong policy focus on technological development nationally, setting out a direction to promote robot development and dissemination (41–43). The robot density in Japan ranks 3rd in the world (390 units) after South Korea (932) and Singapore (605), and Hong Kong comes 6th (275) (38). China, to which Hong Kong SAR belongs, became the country with the largest operational robot stock since 2016, with 339,970 operational units. This accounts for approximately 20 percent of the total worldwide stock (44). What is more, registered robotics firms in China increased from 221 in 2005 to 6,478 in 2015 (44). While robot production and adoption in China is primarily concentrated in manufacturing, expansion is closely linked to government policy, such as the “Made in China 2025” policy, which included subsidies (44). In Japan, the “robot care equipment five-year development plan” was mentioned as part of the Japanese government’s Revitalization Strategy in 2013 (Prime Minister’s Office of Japan, Headquarters for Japan’s Economic Revitalization, 2014). Subsequently, the Ministry of Health, Labour, and Welfare (MHLW) established the “Care Robot Development and Promotion Office.” In 2018, research into, and development and implementation of care robots were promoted by the Ministry of Economy, Trade and Industry (Ministry of Economy, Trade and Industry of Japan 2018). Furthermore, the Ministry of Internal Affairs and Communications has the Information and Communication Policy Research Institute, which supports research and development from the perspective of smart network robots and ICT innovation. Ethical issues are looked at by this institute. Wright (28) compares and contrasts the Japanese government-led approach to funding and managing research and development of robots with the EU approach, and concludes that they differ considerably in their policy priorities and commercialization practices.

Therefore, it could be argued that top-down, state-led development and investment in robots in East Asia can be contrasted with supranational (i.e. EU) level research-led robot innovation in Europe.

### 1.3. Hopes and fears regarding care robots

When it comes to care robots, current use worldwide is still deemed limited. The general public’s attitudes towards robots and their acceptability can largely be influenced by the way they envision their future care and how potential risks are portrayed in the media and in the literature. Above all, the mobilization and investment of researchers, care professionals, industry or policymakers - actors who are key in the production and diffusion of discourse on care robots - also directly respond to the expansion and embedding of these technological promises in society. For several decades, a growing body of literature in social sciences has analyzed how healthcare policies and practices are framed by visions of the future, and has underlined the performative nature of these visions in a modernity marked by the coproduction of science, technology, and society (45,46). Different conceptualizations of these anticipations have been proposed: hope (13), expectations (47,48), promise (14,49) or “sociotechnical imaginaries” defined as “collectively held, institutionally stabilized, and publicly performed visions of desirable futures, animated by shared understandings of forms of social life and social order attainable through, and supportive of, advances in science and technology” (50).

Envisaging robots within this framework therefore allows us to engage with a comparison that can be conducted both between space and time. We can investigate cross-national/cross-cultural similarities and differences in our responses to new and emerging technologies, while highlighting the national or regional patterns of impact of collectively-held future visions on research and development and public policies. Conducting such an analysis of the media contents is all the more relevant, as Jasanoff points out, because the alliance between the media and corporate interests will “play a pivotal role in making and unmaking global sociotechnical imaginaries” (50).

When disruptive technologies are discussed, utopian discourses often co-exist with dystopian ones and - as Kitzinger and Williams (51) have shown in the case of embryo stem cells - the combined rhetoric of hope and fear is used to maximum impact in the media. The discourse on the technological progress of care robots therefore carries a mixed sense of hope, promise and fear, tilting towards the former in public discourse. The “promise” of social robots described in the media and scientific literature includes effective therapy, companionship and social facilitation in cognitive training and physiological therapy (52). The incarnation of this promise (49), through numerous representations of rejuvenated and vibrant older people interacting with robots, further strengthens the promising features of social robots. Even when critical voices are raised relating to ethical issues (protection of privacy) or social (dehumanization, robotization of social relations), this does not destroy the promise completely. On the contrary, the effect of criticism is to give substance to the promise, to make it more credible, to give it an apprehensible social form and to stress the inevitability of the future (53). The ethical urgency only normalizes the promise of robots. It can be argued therefore that there is a looping effect, which transforms any critical discourse, especially on an ethical level, into actions which are the foundations of a technological revolution.

As the global pandemic provided the opportunity for service providers and users to experience the merit of using care robots, promissory discourse on robots has gained new visibility and could well become a “critical juncture” (54) for the development of human-technology interactions in care settings.

With this in mind, it is very timely and meaningful to look back at the trends in public discourse concerning care robots in different jurisdictions across East Asia and Western Europe. The discourse that is ubiquitous, and therefore is expected to appear in all countries’ news media are: population aging, the shortage of care workers, and techno-globalism (against, for example, the background of Sino-American hegemonic rivalry). According to the UN’s report in 2019 [(55), p. 5], by 2050, one in six people in the world will be over the age of 65, up from one in eleven in 2019. All societies in the world are in the midst of this longevity revolution. With these demographic changes, the shortage of care professionals has been raised as an urgent public policy issue by various actors and organizations (56,57). Technology use and digitalization of care are often portrayed as potential solutions to the two issues (population aging and the lack of care workers) (30). Subsequently, with a spread of reasonably-priced, AI-powered and IoT-enabled appliances such as Alexa and smartphones across the globe, people’s lives, connected with digital technologies, have become increasingly borderless. While this trend of techno-globalism enables R&D collaboration in the scientific community for finding solutions to global aging, it could also mean that the competition for technological advancement among companies and national jurisdictions (techno-nationalism, as previously mentioned) can become even more fierce.

Selecting four countries and one region (France, Great Britain, Ireland, Japan and Hong Kong Special Administrative Region of the People’s Republic of China (hereafter, Hong Kong SAR)), this article addresses the question: “how are care robots described in the public domain in Asia and Europe?.” Two subsets of questions are: (i) What are the general trends in newspaper articles that deal with care robots (e.g. frequency of reporting)?; (ii) What are the hopes and promises associated with care robots and what are the concerns?; and (iii) Are there any geographical/national characteristics (e.g. techno-nationalistic discourse) that connect discourse and policy/research and development?

So far, there is a body of literature showing findings from cross-sectional studies (interviews and questionnaires from one single or several selected countries), in addition to the literature review sourced primarily from articles written in English. There is a dearth of research looking at medium- to long-term trends, and cross-regional comparison spanning different languages. Little research has been conducted with a multidimensional focus (descriptions of care robots, research and development process, policy and regulation regarding care robots, and their impact on people and society), particularly with regard to countries where languages other than English are spoken. An international research team was therefore convened, bringing together interdisciplinary researchers (public policy, sociology, social work, nursing, psychology, medicine and medical engineering) from four different jurisdictions (France, Hong Kong SAR, Ireland and Japan).

## 2. Methods

### 2.1. Study design

In order to address the questions, coverage in the printed media was used as the most reliable comparative data. The printed media have a relative advantage over a systematic review of literature for such cross-cultural comparative analysis, as the printed newspapers have long tried to mirror the opinions of the general public, more broadly and impartially, though often from certain political perspectives (58,59).

We initially conducted a document analysis, sketching out each jurisdiction’s government policy and professional guidelines on robotics research and its practical use (60). Then we selected one broadsheet newspaper from each of five jurisdictions, covering four languages (Chinese, English, French and Japanese). Given the small circulation of the Irish newspaper, a British newspaper was added to the list. These five jurisdictions are all unitary and centralized states, unlike federal states such as Germany or the United States, and therefore, each “national” broadsheet newspaper can be representative of the jurisdiction. In addition, using the above-mentioned robot density figures (38) as a proxy, the sample of these five jurisdictions has a good spread, with low and high robot production levels.

The five newspapers were: Le Monde (France), The Times (Great Britain), Ming Pao Daily News (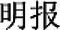 in Chinese, Hong Kong SAR), The Irish Times (Ireland) and Yomiuri Shimbun (
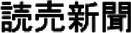
 in Japanese, Japan). These five broadsheet newspapers were selected based on the high volume of circulation in each jurisdiction, combined with a relatively high level of trust in the international community and the data availability covering the period.

Subsequently, a keyword search was conducted, using the following words for each language [French: robot* and (vieillissement | “personnes âgées” | soin | démence | Alzheimer); English: robot* and (ageing | “older people” | care | dementia | Alzheimer); Chinese: 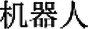
* and (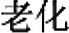 | 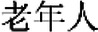 | 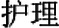 | 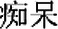 | 
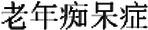
); Japanese: 
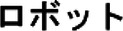
* and (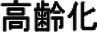 | 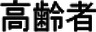 | 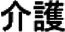 | 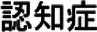 | 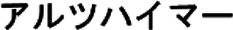)].

The period covered was between January 2001 and September 2020 (19 years 9 months). Once the articles were collated, at least two members of the team checked the articles, and only those referring to “robots” and “older people” or “care” were retained while the rest were excluded. This study was conducted with a particular focus on care robots as a form of assistive technology, referring to robotic assistance used in older people’s care. It is, however, presented within the context of the UNCRPD and implications for disability and rehabilitation policy and independent living (61). The term “care robot” is defined here as a general expression for devices and systems that perform functions such as monitoring of care recipients and their surroundings, and provision of support for care recipients and/or their caregivers (including communication that enables interactive conversation, assistance with activities of daily living, or managing medications) (8). The English translations were produced or checked by native speakers on the research team. The basic profile of each jurisdiction covers its population, demographics and government’s policy in AI/robotics (Table 1).

**Table 1:** Profiles of five jurisdictions.

Region	Western Europe			East Asia	
Jurisdiction	France	Great Britain	Ireland	Hong Kong SAR	Japan
Population	67,063,703	66,796,800	4,977,400	7,474,200	126,167,000
	(2020)	(2019)	(2020)	(2020)	(2019)
Proportion of older people (aged 65 or older)	20.5% (2020)	18.5% (2019)	14.5% (2020)	19.1% (2020)	28.4% (2019)
Key policy documents for robotics/AI (particularly for use by care, personal and welfare services)	No real policy in relation to robots.	The Industrial Strategy Challenge Fund (ISCF) was created within the government’s Industrial Strategy to ensure that the UK’s strengths in research and innovation deliver even more tangible results with economic and public benefits. The fund will support challenges on a distributed and local basis, helping to incentivize innovations and solutions to issues that may not be funded in the private sector without government encouragement, but are of high public value, for example: Improving the efficiency of social care provision by actively mapping capacity, logistics demand and forecasting.	There has not yet been government-wide coordination around AI and robotics in connection with health or social care.	In Hong Kong SAR, the government invested one billion (HKD) in Dec. 2018 to establish the “Innovation and Technology Fund for Application in Elderly and Rehabilitation Care.” The aim of the investment is to subsidize elderly and rehabilitation service units to procure, rent and trial technology products, so as to improve the quality of life for service users as well as reduce the burden and pressure on care staff and carers. The fund has opened two rounds of application and has granted about $140 million to subsidize 770 elderly and rehabilitation service units to procure or rent over 2,900 technology products.	In Japan, under the auspices of the government, the development of care robots is moving forward with the aim of achieving Society 5.0.
	In 2007, the robotics GDR (Research group) was created in CNRS (the National Centre for Scientific Research).	“Robotics in Social Care: A Connected Care EcoSystem for Independent Living” published by UK Robotics and Autonomous Systems Network (2017).	However, the Department of Health initiated a blue skies policy project looking at the potential for AI and robotics to transform the health workforce and health services. This reflects the government’s commitment shown in the national health reform programme “SláinteCare” which was published in 2018.	In 2020, Legislative Council of HKSAR issued “Application of gerontechnology in elderly care services”. (Legislative Council of the Hong Kong Special Administrative Region of the People’s Republic of China 2020).	Cabinet Office published the “New Robot Strategy” (2015).
	In 2013, “France Robots Initiatives plan” published		At European level, the European Commission published a report “Ethics Guidelines for Trustworthy Artificial Intelligence” in April 2019.		In 2015, the MHLW introduced a subsidy of 100,000 yen per device to allay costs for insured care facilities in introducing care robots.
	In 2017: Report on behalf of the Parliamentary Evaluation Office, Science and Technology Choices, “For mastered, useful and demystified artificial intelligence” published.				Funding schemes began with the Japan Agency for Medical Research and Development and the METI for pilot studies on care robots with communication and social support functions for older people.
	The publication of “Artificial intelligence and work” and “Digital and Health Which Ethical issues for which regulation?” (CCNE 2020).				The “AI Network Society Promotion Council” established by the MIC in 2016 (MIC n.d.).
	President Macron presented France’s “AI Strategy” (France Stratégie 2018) Network (2017).				In 2018, the Office for Nursing Care Robot Development and Promotion established in the MHLW.

(Demography: INSEE n.d.; Office for National Statistics n.d.; Central Statistics Office, Ireland. n.d.; Statistics Bureau of Japan. n.d. Census and Statistics Department, The Government of the Hong Kong Special Administrative Region. n.d.)

## 3. Results

### 3.1. Analysis of trends

In total, the number of articles retained for further analysis was 545 for the period between January 2001 and September 2020. Out of these, the breakdown for each jurisdiction of France, Great Britain, Ireland, Hong Kong SAR and Japan was 94 (17.2%), 71 (13.0%), 25 (4.6%), 74 (13.6%) and 281 (51.6%), respectively. More than half of the total number of newspaper articles originate in Japan, and the smallest ratio was from the Irish newspaper.

**Figure 2:**
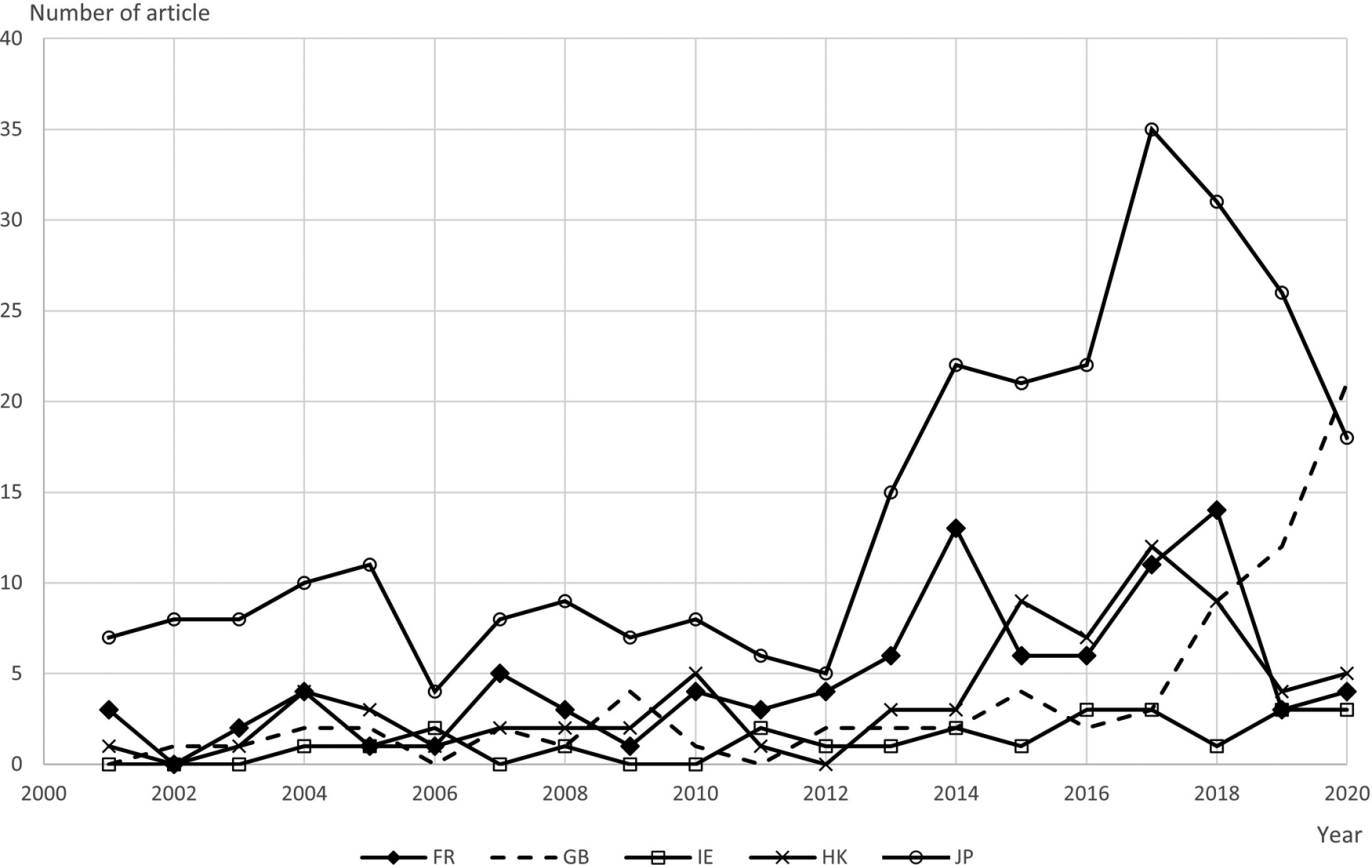
Number of newspaper articles by jurisdiction, year-on-year change, January 2001 – September 2020.

The yearly trend of each jurisdiction’s newspaper can be found in Figure 2.

The British newspaper shows a steady increase since 2017, while the French and Hong Kong newspapers indicate peaks and troughs since 2012. A consistently low number was found in the Irish newspaper, and a great increase in the number for the Japanese newspaper in 2013 and 2017, followed by a reversal of the trend after 2018.

Considering that more than half of the entire collection derived from Japanese newspapers, this affects the overall pattern. However, between January 2001 and September 2020, there are two critical junctures, judging from Figure 2. The first one is 2007, and the second is 2012/13. The mid-2000’s coincided with an increase in investment into and research funding of robotics across Europe (e.g. the 7th Framework Programme, 2007–2013). In Japan, the then Prime Minister published the “New Robot Strategy” in 2015, increasing government investment in the area.

### 3.2. Themes

**Figure 3:**
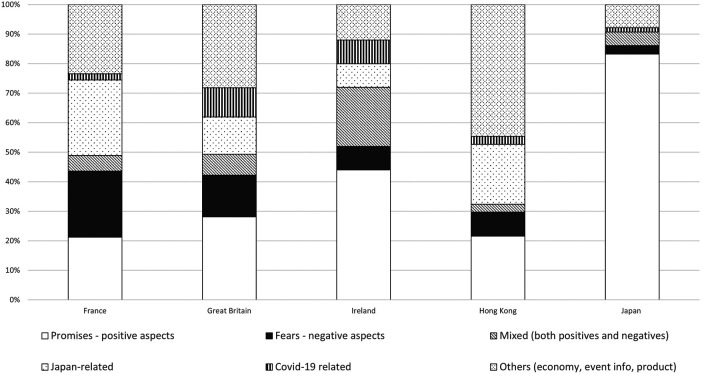
Six-way classifications of the newspaper articles collected from the five jurisdictions.

Further analysis was carried out, and the following six categories were identified (Figure 3). These are: positive aspects (promises and policy solutions), negative aspects (fears, concerns and ethical issues), mixed views (both positive and negative), Japan-related news, Covid-19 related news, and other news (e.g. event info, products). Below, the negative aspects and mixed views are combined into one section.

#### 3.2.1. Promises and policy solutions

Generally, the findings show that a lot is promised when robots are mentioned. Robots, because “they are our future,” are presented as a major economic stake and often as public policy solutions to an aging society. The future seems to lie with the robot industry, which is a prominent feature of many Japanese newspaper articles, although this phenomenon is not restricted to Japan.

In an article entitled “Our dream is (to live in) a robotics nursing apartment” (62), one entrepreneur speaks about his plan to make his locality a hub for the robot industry (Table 2).

**Table 2:** Illustrative quotes for five themes.

Themes	Illustrative quotes
Positive views	“Although robots cannot replace people, with robots’ help, one person could do what normally requires the manpower of three people (…) using robots will create a society where people can support each other and protect our human dignity.” (62).
	“Aging at home in 2030. Robotic aids, remote assistance, smart homes - progress in technologies and the housing of tomorrow should make older people less dependent and less lonely” (63).
Negative views	“In some nursing homes, pets are already starting to be replaced by plush therapy robots - the idea being to provide affection to the elderly without the expense. Likewise, there will undoubtedly one day be robots to replace guide dogs, or even sheepdogs. What do we have to lose? Intelligence. When a dog guides sheep, he obeys specific instructions, but he organizes things in his own way: we rely on his intelligence while he relies on the trust that we have in him, and this relationship itself makes us smarter. Nothing like that with robots!” (64).
	“Robotics run the risk of dehumanizing working relationships and assisting people, in hospitals for example, and will pose ethical problems. Don’t these techniques run the risk of massively destroying jobs? … The fact remains that progress at the frontier of neurosciences and information technologies will pose the human-machine relationship in new terms. This societal issue deserves in-depth reflection, in particular on the serious risks of control by a few companies, especially multinationals, and administrations of the techniques and data that they will handle…” (65).
Mixed views	“A laboratory in Vancouver is advertising for staff to help robots to work with people with developmental disabilities. The robot will learn to recognise ‘various movements and activities, such as sitting, standing, laying on the floor, exercising, eating, etc., to better track the progress of residents in a group home.’ If our child has a developmental disability, he might very well find himself interacting with one of these robots as a normal part of his life. There is, of course, a dark side. Will this child, when he grows up, have a job to go to? There seems little doubt at this stage huge swathes of jobs are going to be taken over by AI. Will he be dependent on some sort of basic income provided by the State?” (66).
Japan-related articles	“Robotics, less expensive than home help, will help people in their daily tasks: housework, walking assistance, etc. However, fake humans or fake pets are also thought to serve emotional needs. The Japanese thus tested a baby seal, Paro, who moves, cries or is joyful, reacting to the emotions of his interlocutor” (67).
COVID-19-related articles	“Although the lady was nearly blind, her family could see her healthy condition from her face, thanks to the video calls” (68).

This type of narrative is common, straddling local economy development and age-friendly community, with robots playing their roles.

There are many different types of robots mentioned in the Japanese articles, ranging from Aibo, Pepper, Toyota Partner robots (e.g. a robot that plays violin), Paro and HAL (Hybrid Assistive Limb) to RIBA (which can lift up or set down a human being from or onto a bed or wheelchair). Expos and special events featuring robots are also featured in these newspaper articles. From industry to market and communities, the all-nation approach is clearly seen, both in the volume and range of the information provided in the Japanese newspapers, which was not the case in the other four jurisdictions’ newspaper articles.

Much promise often stems from scientific evidence or technological innovation, potentially leading to successful commercialization in the market. It is not only in Japan where robots’ various assets were described as the vision for future care. As early as 2006, the Irish newspaper reported on various kinds of robots that had been in use in hospitals in the United States. Robots named the Tug, HelpMate and RoboCart roll around hospitals dispensing medications to nursing stations, while in the Mayo Clinic in Minnesota, RoboCarts were reported to carry blood samples to labs (69). The European Union’s research project Redeem brought a home care robot called Giraffe, made in Sweden, into a remote area of Scotland (Western Isles) for testing (70). The robots were designed to help people with dementia live independently. In France also, a discourse promising much with regard to robots was very visible in newspapers, sustained by interviews with innovators and entrepreneurs, and was increasingly linked to a more general hyperbolic vision of AI possibilities.

#### 3.2.2. Fears, concerns and ethical issues

“An ethical approach” is mentioned by the Irish Times (71), citing a researcher at the Georgia Institute of Technology in Atlanta, the United States.

“Robotic systems are close to being pervasive, with applications involving human-robot relationships already in place or soon to occur involving warfare, childcare, eldercare, and personal and potentially intimate relationships.” (71).

In the two newspapers in Europe, the causes for concern included the risk of illusion, confusion and then the weakening of human-to-human ties and human-to-animal connections. Another article warns that scientific progress today can weaken society tomorrow (Table 2).

From the human rights perspective, questions were raised (e.g. “what are the limits of the new technologies?,” “do we want the care of some of the most vulnerable citizens to be undertaken by a machine because it is cheap?”) (72).

The Irish Times articles were most balanced, in that they often portrayed robots both positively and negatively (Table 2).

#### 3.2.3. Japan – the land of robots?

Overall, it can be argued that Japan is regarded as both the gold standard and a preview of the future in terms of robots, despite the fact that research into robotics is also active elsewhere, in China, Germany and the USA. Many articles referring to Japan were found in the newspapers from France, Great Britain, Ireland and Hong Kong SAR.

Other countries and regional blocks that were mentioned in the articles collated include: USA, China, Singapore, South Korea, Germany, Denmark, France, Great Britain, Brazil, Oman, Saudi Arabia, the European Union and ASEAN. Japan was most frequently cited in all five newspapers.

Some of the robots mentioned as originating in the USA or Great Britain are worth noting. For example, Atlas was built by the robotics company Boston Dynamics for the Defense Advanced Research Projects Agency (DARPA), in the United States. An article in The Times records what happened to the robot developed by Japan’s large manufacturing firm Toshiba for the purpose of removing radiation debris in the aftermath of the Great East Japan Earthquake. The robot, which was sent into the danger zone, failed to perform decontamination work during its demonstration, and the Tokyo Electric Power Company, owner of the plant undergoing decontamination, had to turn to Great Britain’s QinetiQ, and iRobot of the United States (73). The involvement of French company Aldebaran in putting together the humanoid robot Pepper (originally manufactured by Japan’s SoftBank) hit the headlines in France (74).

What is unique about Japan is that the country is perceived as a global hub and laboratory for aging and its possible “robotization.” The population is described as culturally permeable to these new technologies and their introduction into private life, although in reality this may not be true. In Japanese newspapers, robotics is not discussed purely as a tool or an instrument but as “something” to co-exist and interact with human beings in society.

In Le Monde, a connection was even made between robots and longevity. Human longevity seems inseparable from the progress of robotics. The robot is envisioned both physically and emotionally: these are the first evocations of Paro (Table 2).

#### 3.2.4. COVID-19 related news

One of the potential critical junctures is the COVID-19 pandemic, which put the use of technology for care in the spotlight. In Hong Kong SAR, the video-call installed robot helped connect older people with their relatives during the pandemic (Table 2). One residential facility introduced a robot called TemiMedic in September 2019, which enabled entry into the isolation room without fear of infection for the staff. The hospital arranged for one family to talk to their grandmother twice a week through the video call function of TemiMedic.

Similarly, positive narratives around the use of robots began to emerge in all jurisdictions and are expected to continue.

#### 3.2.5. Other themes, including care robots for people with disabilities

Reflecting on the lack of robot-specific policy and guidelines in many countries, apart from Japan and Denmark, it becomes clear that different jurisdictions are at different stages of research and development. In 2018, the Hong Kong SAR government introduced the Innovation and Technology Fund for Application in Elderly and Rehabilitation Care (Table 1), to provide financial support for older adults and rehabilitation centers to buy or rent gerontechnology products. According to the updated figures, the Hong Kong SAR government has received 1600 applications from 450 service centers and approved 1100 applications. The founder of a local start up stated that some of the applications were approved within two months because of the pandemic. However, some of the applications will still need to wait for a year for approval, so he wishes that the government can speed up the approval process to meet the rising demand of gerontechnology, especially for robots with video call function, during the pandemic (75).

In the British and Irish newspapers, the policy-related articles tended to be focused on the economy, jobs and the market (71,72). Similarly, in the French newspapers, when the national agency for vocational guidance France Compétences was established, an article highlighted increasingly fierce competition between human beings and robotics/AI in various sectors, and how the French government sought to provide support and upskilling opportunities (76). In addition, other policy adaptations to the aging society and potential market growth for the silver economy were also indicated, with ministers and politicians often being quoted (74,77).

On the other hand, in the Japanese newspaper, there is a plethora of policy-related articles, dating back to 2003. They are often linked to regional development plans and industrial policy to promote innovation in research and care facilities. “Osaka City also plans to set up a development base in a redevelopment area near JR Osaka Station, and many local governments such as Kanagawa Prefecture and Kobe City are working to foster the robot industry” (78). There was even a parliamentary candidate who made reference to care robots at one general election.

“With the aging society and the declining labor force, it is necessary to introduce care robots. It will soon be available for home care,” said a former Liberal Democratic Party member in the 3rd district of Ehime Prefecture (79).

The milestone for Japan came in 2015 when the government published a “New Robot Strategy.” In this document, robots were presented as multi-purpose, playing a variety of roles in society on behalf of human beings, as a response to population decline. The goal was to increase the number of jobs that use robots in fields such as social and healthcare, and to expand the domestic robot market in 2020 to 2.4 trillion yen, which was four times greater than that of 2015 (600 billion yen) (80).

Among the articles collected for the analysis, 65 Japanese articles showcased or mentioned care robots for people with disabilities, while 7 Hong Kong, 5 French, 4 British and 2 Irish newspaper articles featured them. Although the majority of the articles were focused on the functionalities and R&D aspects, some recent articles began to highlight (potential) positive outcomes of these care robots such as increasing social inclusion and combatting loneliness (81).

“OriHime is a ‘social participation type avatar robot’ that allows people who cannot move due to illness or disability to meet people they want to meet and go to places they want to go. A person on the bed can remotely control the robot with a smartphone or other device, and use the camera and microphone built into OriHime to have a conversation with people nearby and see the surroundings as if they were there.” (82).

### 3.3. Further analysis of trends and cross-regional comparisons – Western Europe vs. East Asia

**Figure 4:**
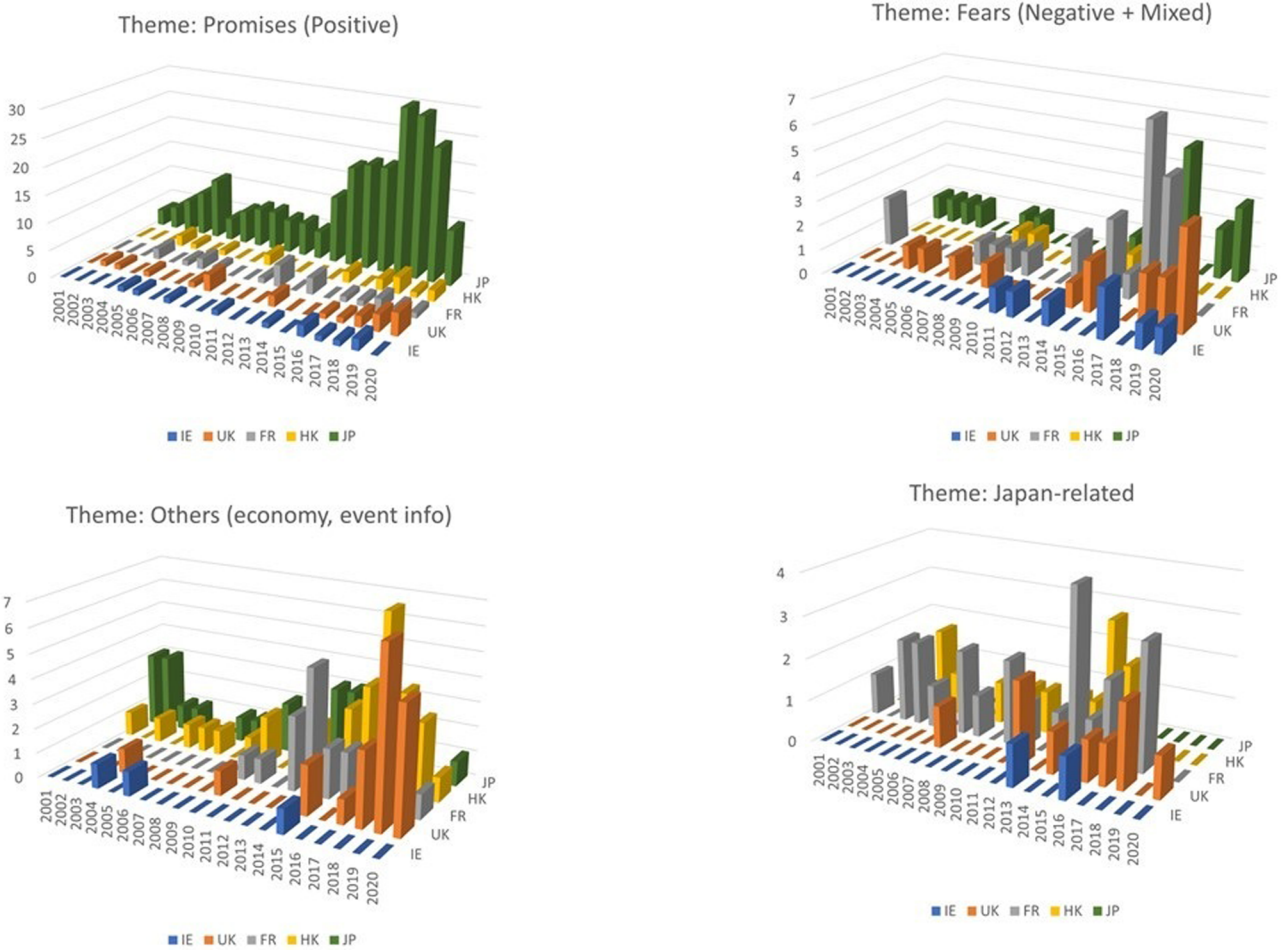
Thematic 3D comparison for five jurisdictions over time.

Figure 4 shows four thematic 3D comparisons of the articles from five jurisdictions over the period. A relatively strong presence of negative or mixed views was found. Comparing the five jurisdictions’ newspapers, the “fear” and “concerns” described were where the most distinct difference between Western Europe (particularly Britain and France) and East Asia was found in the way in which robots are portrayed in newspapers (Figure 3).

The British journalist Will Humphries wrote that

“they will ‘turn evil’ and steal everybody’s jobs. The Japanese and Americans cannot wait to be pampered by them. (…) The vision is welcomed with open arms in the Far East and USA but robot developers at Honda say Europeans, and especially the British, are far more wary” (83).

The Hong Kong newspaper cited public opinion in the USA and Europe concerning care robots, explaining why there was still some resistance to them. The Director of the Mechanical Center of the University of Edinburgh was quoted as pointing out that

“the resistance to care robots may stem from the fear that elders who receive ‘non-human’ care would become more isolated, and the robots collect personal data which may also cause privacy issues.” (84).

These types of ethical concerns are very rarely recorded in Japanese newspaper articles. In contrast, a company CEO in Japan was quoted as saying that

“what is worrisome is the overwhelming shortage of engineers in the country. I feel that not only our company but also the companies we sell our products to have a shortage of engineers who can understand how to incorporate robots into factories and improve manufacturing efficiency.” (85).

A concern was also expressed about the technological promise that creates “the blind dynamic of technological growth.”

“While we are already totally overwhelmed by the combined effects of the consumer society, is there any sense in funding research aimed at surrounding us with ever more machines? Robots in the homes of all dependent people, and in all hospitals?” (86).

Concerning the same issue, one interpretation recorded in the Japanese newspaper is different. The above-mentioned Professor Ishiguro (Osaka University) commented that

“the background to the attention paid to robots is the decrease in daily communication (…) Spending more time on computers and smartphones has led to reduced conversations. Some people are shy and cautious about talking to other people, and there are many older people living alone (…) With a robot, you can talk with no hesitation, and it’s easy to share one’s frustration and anxiety” (87).

The logic here is the reverse of that expressed in the British and French newspapers.

The period 2012/13 is a critical one for the increased attention. Given that French, British and HK papers’ interest in Japan has been relatively constant, once care robots have become more of a domestic issue, in France and Britain, split opinions began to emerge, while in Hong Kong, the articles were more concerned with the impact on economy and publicity of robots without much critical analysis, similar to Japanese newspapers.

## 4. Discussion

In addressing the question: “how are care robots described in the public domain in Asia and Europe?,” we found that newspaper articles often described robots positively, as something that can offer us hope and much promise. In this sense, technological determinism is somewhat strong in the newspaper articles. However, this is no surprise, as these reports had to be “newsworthy” for them to be printed in a major broadsheet newspaper in the first instance. The publications regularly highlighted new discoveries and novel functionalities found and developed, both in their own jurisdictions and abroad.

By breaking down the collection of articles into the six categories, we were also able to capture changing trends and patterns. A general upward trend in the number of articles on the subject was observed for 2012 onwards in most jurisdictions, with articles increasingly beginning to focus on the impact of care robots on human beings and society. The emphasis on these aspects of people and society was stronger in Western European than in East Asian cases for this study (Figure 4).

The fact that Japanese newspapers accounted for more than half of the entire collection corresponded to the fact that many articles in other jurisdictions looked at Japan as the land of (care) robots. This is a good example of how certain technologies are culturally and socially embedded, and the image of Japan as a robotics-friendly country was certainly reproduced through these print media (in Japan as well as other countries). The public discourse around care robots in Japanese newspaper articles was further reinforced by the Japanese government’s dedicated policy and strategy, and this is where a social constructivist (as opposed to technological determinist) approach to the “technological frame” (9) can be witnessed.

The concept of “technological frame” consists of three major themes: (i) the nature of care robots (potential users’ images of care robots, and their understanding of care robots’ functionalities and capacities); (ii) care robots in use (potential users’ understanding of how they will use care robots in care work); and (iii) care robot strategy (potential users’ understanding of why care robots are procured or deployed in care, and the expected outcomes and values from the organization’s perspective). Therefore, the sources of perceptions can be more complex and multi-layered. When the sales figures of industrial robots are examined, it can be seen that they grew by nearly 30% in 2014 (approximately 230,000 robots sold worldwide). While the level of growth in robot utilization in manufacturing has been rather dramatic in China and the USA since 2012, that of Europe has been modest (+4% growth per year) (88). Amid this situation, the perception that Japanese people are friendly towards care robots persisted in newspapers in Europe. In Japan, on the other hand, as Wright observes (28), the government-led approach to funding and managing research and development of robots appears to have permeated its domestic print media, and the same can be said of the Hong Kong articles. This explains the high proportion of articles referring to economy, expos and special events featuring robots in Hong Kong.

One of the interesting findings here is that there seems to be a diffusion of ideas crossing national borders. There certainly is a socially constructed idea of care robots, described in these media, and that generates further interest in new products (social construction of technologies). However, on top of that, the number of French and British articles trailed the Japanese ones, and when robots started to become a “reality” in their own jurisdictions, their analysis became more nuanced. The ethical dilemma of using care robots was one of the universal themes, as was concern over carers losing their jobs. The fear of robots replacing human beings’ jobs, on the other hand, partially stems from inadequate policy measures addressing underfunded social care and the lack of coordination among policymakers, R&D researchers, care professionals and citizens (89–92). This is where the “technological frames” (the nature of care robots, care robots in use and care robot strategy) matter greatly (93). Different types of care robots (physical support type and socially assistive type) interact differently with users and therefore require different approaches and strategies at different levels in society. Such a fine-grained analysis of social robotics was hardly discovered in the newspaper articles. It is worth noting however that amongst many care robots that have been developed, those introduced into rehabilitation and telework for people with disabilities began to provide concrete examples of their usefulness not only for users themselves but also for wider society. There is great potential for achieving a more inclusive society if care robots are appropriately implemented and used to empower users and their carers.

This research has several limitations. We only used a single source from each jurisdiction, and there could be a greater variety of data if we collected it from multiple newspapers. The fact that Ireland had only a few articles does not necessarily mean the general public has no interest in care robots in Ireland. In fact, as previously noted, Stevie the Irish robot made the cover of Time magazine. In addition, the differences in reporting styles between Western Europe and East Asia can be ascribed to different expectations for broadsheet newspapers’ roles in each society. Because of the selected number of jurisdictions (the UK, France and Ireland from Western Europe and Japan and Hong Kong SAR from East Asia), a sweeping generalization is not appropriate. Despite these limitations, the medium-term trend of public discourse around care robots in the five jurisdictions in Western Europe and East Asia was captured, generating interesting findings. Future fieldworks are required, using a wider variety of documents, images and artifacts beyond the analysis of newspaper articles. Further research such as interviews and focus groups with key stakeholders should also be undertaken in order to understand their views of and experiences with care robots in different cultures.

The worldwide COVID-19 pandemic was very transformative and unique, as in many instances, technology became almost the only method of connecting people both within local communities and across the globe, and has been a tool in providing support for vulnerable groups in society, such as those with chronic conditions and disabilities, and older people (4,94). Wearable electronic devices, for example, were used to capture physiological signals in the early detection of asymptomatic and pre-symptomatic cases of COVID-19 (95). The scope for the use of WTs dramatically expanded, and this was a global phenomenon. Telemedicine and telecare began to be embraced, as they have the potential to enable people to stay and age in their community. The pandemic accelerated the change that was already envisioned and happening, although it opened the door to trialing something that had previously been deemed unsuitable for care settings. The service robotics industry has indeed grown during the pandemic, and the trend is expected to continue (96). Care robots would not be the exception. A recent survey carried out in Finland demonstrated that conventionally negative views held by eldercare professionals toward telecare robots took a positive turn during the pandemic (97). It remains to be seen whether this trend is observed across Europe.

## 5. Conclusions

This article explored how care robots have been described in two jurisdictions in East Asia and three countries in Western Europe. It found more positive or uncritical views towards care robots in the newspaper articles in East Asia than in Western Europe. In addition, a much stronger sense of “technological determinism” was identified in Japanese newspapers, while the newspaper articles outside Japan continued to portray the country as the land of robots. Globally, the competition over technological advancement in robotics and AI is becoming fierce, and the care sector across most countries is in dire need of resources. There remains a mismatch between a future vision of care and what is available. As care robots begin to be used and portrayed as part of technological solutions to fill this gap, interest in these welfare technologies is increasing and diversifying. The rhetoric of a “robot revolution” has become pervasive, creating both hype and fears for the care sector, the labor markets and society at large. Whilst the impact of using care robots is yet to be known and scientifically evaluated, certainly the pandemic has accelerated their use, and further careful examination of various types of care robots in rehabilitation will be even more necessary beyond national and cultural boundaries in the future.

## Data Availability

The datasets used and analyzed during the present study are available from the corresponding author on reasonable request.
